# Characterization of Acid Sphingomyelinase Activity in Human Cerebrospinal Fluid

**DOI:** 10.1371/journal.pone.0062912

**Published:** 2013-05-02

**Authors:** Christiane Mühle, Hagen B. Huttner, Silke Walter, Martin Reichel, Fabio Canneva, Piotr Lewczuk, Erich Gulbins, Johannes Kornhuber

**Affiliations:** 1 Department of Psychiatry and Psychotherapy, Friedrich-Alexander-University of Erlangen-Nuremberg, Erlangen, Germany; 2 Department of Neurology, Friedrich-Alexander-University of Erlangen-Nuremberg, Erlangen, Germany; 3 Department of Neurology, University of the Saarland, Homburg/Saar, Germany; 4 Experimental Therapy, Franz-Penzoldt-Center, Friedrich-Alexander-University of Erlangen-Nuremberg, Erlangen, Germany; 5 Department of Molecular Biology, University of Duisburg-Essen, Essen, Germany; MUSC SC College of Pharmacy, United States of America

## Abstract

**Background:**

As a key enzyme in sphingolipid metabolism, acid sphingomyelinase (ASM) is involved in the regulation of cell fate and signaling via hydrolysis of sphingomyelin to form ceramide. While increased activity of the lysosomal form has been associated with various pathological conditions, there are few studies on secretory ASM limited only to cell models, plasma or serum.

**Methods:**

An optimized assay based on a fluorescent substrate was applied to measure the ASM activity in cerebrospinal fluid (CSF) collected from mice and from 42 patients who were classified as controls based on normal routine CSF values.

**Results:**

We have detected ASM activity in human CSF, established a sensitive quantitative assay and characterized the enzyme’s properties. The enzyme resembles plasmatic ASM including protein stability and Zn^2+^-dependence but the assays differ considerably in the optimal detergent concentration. Significantly increased activities in the CSF of ASM transgenic mice and undetectable levels in ASM knock-out mice prove that the measured ASM activity originates from the ASM-encoding gene *SMPD1*. CSF localized ASM activities were comparable to corresponding serum ASM levels at their respective optimal reaction conditions, but no correlation was observed. The large variance in ASM activity was independent of sex, age or analyzed routine CSF parameters.

**Conclusions:**

Human and mouse CSF contain detectable levels of secretory ASM, which are unrelated to serum ASM activities. Further investigations in humans and in animal models will help to elucidate the role of this enzyme in human disease and to assess its value as a potential biomarker for disease type, severity, progress or therapeutic success.

## Introduction

Enzymes involved in sphingolipid metabolism influence the concentration of bioactive lipids such as ceramide and sphingosine-1-phosphate, thereby controlling diverse cellular processes including proliferation, differentiation, migration, adhesion, growth arrest and apoptosis [1]. Among these enzymes, acid sphingomyelinase (ASM, EC 3.1.4.12) is a lysosomal glycoprotein that catalyzes the hydrolysis of the major eukaryotic membrane constituent sphingomyelin (SM) to yield phosphorylcholine and ceramide. Ceramide functions as a messenger and a structural component that participates in receptor clustering, vesicle formation and fusion [2]–[4]. Ceramide can be further degraded by ceramidase to form sphingosine which is subsequently phosphorylated to sphingosine-1-phosphate, an intracellular and extracellular lipid mediator and a potent mitogen [5]. In this context, the regulation of sphingomyelinase and ceramidase activities is responsible for the dynamic balance between ceramide and sphingosine-1-phosphate; since these two lipids often act antagonistically on the cell’s fate, the system is referred to as a “rheostat” [5], [6].

Independent genes encode several species of sphingomyelinase, which differ in their optimal activity at acidic, neutral or alkaline pH. The gene *SMPD1*, which encodes ASM, has been shown to yield two forms: lysosomal ASM and secretory ASM (S-ASM). Lysosomal ASM acquires zinc ions during trafficking to lysosomes and is therefore independent of additional Zn^2+^. Conversely, S-ASM is either entirely or partially dependent on exogenous addition of Zn^2+^ [7]. Defects in the *SMPD1* gene causing a reduction or absence of ASM activity lead to the rare autosomal recessive lysosomal storage disorder Niemann-Pick Disease [8]. Accumulation of SM predominates in the viscera (Niemann-Pick Disease type B) or can extend to neuronal and glial cells resulting in cognitive deficits, motor dysfunction and blindness (type A). Increased ASM levels have been implicated in various pathological conditions including atherosclerosis [9], major depression [10], Alzheimer’s disease [11], status epilepticus [12] and alcoholism [13], [14].

Although deregulated lipid metabolism may be particularly important for injuries and disorders of the central nervous system, which maintains a high concentration of lipids, brain biopsies are rarely available for studies, and research generally utilizes post-mortem tissues, peripheral blood samples or animal models. Cerebrospinal fluid (CSF) can provide an additional source for information and may contain potential molecular biomarkers. Due to its direct contact with the extracellular space of the brain, CSF may reflect biochemical changes in the brain in response to pathological processes. CSF is therefore routinely collected by a mildly invasive lumbar puncture from patients suspected of having disorders of the central nervous system and examined using various proteomic strategies. Thus far, there have been no reports on ASM activity in CSF. Takahashi *et al.* failed to measure any ASM activity in the CSF of two apparently healthy patients despite detecting significant enzymatic activities in various other human extracellular body fluids [15].

In this report, we provide evidence that S-ASM activity is present in CSF and may be quantified by a sensitive enzyme assay based on a fluorescent substrate. We further characterize the enzyme’s biochemical properties as well as activities in a pilot study in humans and in mice.

## Materials and Methods

### Collection of CSF Samples from Humans

Human CSF was obtained by lumbar puncture from patients to exclude inflammatory diseases of the central nervous system. CSF was collected in polypropylene tubes, centrifuged at 2000×g for 10 min to pellet cells and stored in aliquots at −80°C until use. Twenty patients from the University Hospital Erlangen (group A) were considered as healthy controls based on normal values for CSF color (clear), cell counts (≤4 cells/µl) and total protein (<450 mg/l). In a second study (group B), 22 control samples from the University of the Saarland were used, and corresponding serum samples were also provided (Table 1).

**Table 1:** Clinical data of analyzed study groups with “mean ± standard deviation (median)” and statistical analysis of their influence on CSF S-ASM activity in a general linear model.

				GLM for S-ASM in CSF	
			missing values (A/B)	F	p
study (n)	A (20)	B (22)	–	5.605	0.025
sex (female/male)	10/10	13/9	0/0	0.005	0.943
Age (years)	46.3±18.3 (42.3)	43.9±16.6 (48.5)	0/0	0.953	0.338
CSF cell count (1/µl)	1.65±1.18 (1)	1.48±1.69 (1)	0/1	0.162	0.691
CSF protein (mg/l)	320±87 (294)	366±120 (340)	0/0	1.596	0.217
CSF albumin (mg/l)	210±70 (189)	215±90 (204)	0/0	1.897	0.180
CSF lactate (mmol/l)	1.82±0.36 (1.72)	1.49±0.30 (1.50)	0/4	1.127	0.298
S-ASM activity in CSF (fmol/h/µl)	136±39 (128)	265±124 (232)	0/0	–	–
S-ASM activity in serum (fmol/h/µl)	not available	198±78 (183)	20/0	–	–
plasma CRP (mg/l)	4.4±3.7 (3.1)	23.1±44.1 (3.2)	1/1	1.342	0.257

### Collection of CSF Samples from Mice

Mouse CSF samples (4–7 µl) were collected by puncturing the cisterna magna with a thin glass capillary [16], [17]. Prior to this procedure, animals were anesthetized by intraperitoneal injection of ketamine/xylazine and sacrificed by an intraperitoneally applied overdose of pentobarbital. In addition to monitoring the CSF color, samples were evaluated for blood contamination by detecting released haemoglobin (absorbance at 417 nm on a NanoDrop ND-1000 photometer, Peqlab) following freezing of the remaining sample [18]. Material was collected from ASM knock-out mice [19], mice conditionally over-expressing ASM (E. Gulbins and J. Kornhuber, unpublished), and their wildtype siblings, respectively. The transgenic ASM mice carried the murine ASM encoding gene (*Smpd1*) under the control of the ubiquitous CAG promoter inserted into the *Hprt* locus by homologous recombination (genOway, France). The *loxP*-flanked stop cassette between the promoter and the ASM transgene was removed by mating transgenic mice with mice ubiquitously expressing Cre-recombinase. Heterozygous offspring were used for analysis.

### Ethics Statement

Both human studies were conducted in compliance with the Helsinki Declaration and approved by the Ethics Committee of the Friedrich-Alexander-University of Erlangen-Nuremberg (group A) or the Ethics Committee of the medical association of the Saarland (group B), respectively. The latter granted an exemption for the study on group B to proceed without individual informed consent. All patients in group A gave written informed consent to participate in the study.

Mouse killing was performed in accordance with the German Protection of Animals Act §4 para. 1 and 3. The announcement of mouse killing was approved by the designee for animal protection of the University of Erlangen-Nuremberg (TS-7/12).

### ASM Activity Assay

The activity of S-ASM in CSF was determined using the fluorescent substrate BODIPY-FL-C12-SM (N-(4,4-difluoro-5,7-dimethyl-4-bora-3a,4a-diaza-s-indacene-3-dodecanoyl)sphingosyl phosphocholine, D-7711, Invitrogen/Life Technologies) with 3–4 replicates each. A standard reaction contained 58 pmol substrate (1∶2000 v/v dilution of the 1.16 mM stock solution in DMSO) in a total volume of 100 µl 200 mM sodium acetate buffer (pH 5.0) with 500 mM NaCl, 0.02% Nonidet P-40 detergent and 500 µM ZnCl_2_ and was initiated by the addition of 0.5–2 µl of CSF sample. For optimization of reaction conditions, a series of Carmody buffers (mixtures of 0.2 M boric acid, 0.05 M citric acid and 0.1 M trisodium phosphate) adjusted to pH 3–10 [20], the addition of various detergents or supplementation with unlabeled SM from bovine brain (Santa Cruz, sc-201381) were tested. After incubation for 24 h at 37°C, reactions were stopped by freezing at −20°C and stored until further processing. Next, lipids were extracted by adding 250 µl chloroform:methanol (2∶1, v/v), vortexing briefly and separating the phases by centrifugation for 2 min at 16000 g. The lower organic phase was then concentrated in a SpeedVac centrifuge (Christ Alpha 1–4) for 20 min at 42°C, dissolved in 2 µl chloroform:methanol (2∶1, v/v) and spotted on silica gel 60 thin layer chromatography plates (Macherey-Nagel, 805034). Ceramide and uncleaved SM were separated over a distance of 4 cm using a mixture of chloroform:methanol (80∶20, v/v) as a solvent and were quantified on a Typhoon Trio scanner (GE Healthcare; 488 nm excitation, 520 nm emission, 285 V, 100 µm resolution) with the QuantityOne software (Bio-Rad Laboratories). NBD-C6-ceramide (Invitrogen/Life Technologies, N-1154) and BODIPY-FL-dodecanoic acid (Invitrogen/Life Technologies, D-3822) served as references on the chromatography plates. Enzymatic activity is presented as the hydrolysis rate of SM (fmol) per time (h) and per sample volume (µl).

The activity of S-ASM in serum was determined as described previously [13] with slight modifications: Serum (1.2 µl) was incubated in 100 µl of the reaction mixture (116 pmol substrate in 200 mM sodium acetate buffer (pH 5.0), 500 mM NaCl, 0.2% Nonidet P-40, 500 µM ZnCl_2_) for 24 hours. For comparison, all values were converted to activities with 58 pmol substrate using the factor 0.40 which was determined in separate assays (C. Mühle, unpublished).

For neutral sphingomyelinase activity, the reaction mixture consisted of 58 pmol BODIPY-FL-C12-SM, 200 mM HEPES buffer (pH 7.0), 200 mM MgCl_2_, 0.05% Nonidet P-40 and was performed analogously to the ASM assay.

### Statistical Analysis

Statistical analyses were performed using PASW Statistics 19.0 (SPSS Inc.) applying Student’s t-test and Pearson correlation where appropriate with a significance level of 0.05. Non-linear regression analysis was used for the determination of Km and Vmax values. The general linear model (univariate ANOVA) was applied with S-ASM activity in CSF as the dependent variable, the study group as a fixed factor, and all other factors as covariables. Error bars present in the graphs represent standard deviations for 3 or 4 replicates.

## Results

### Detection of ASM Activity in Human CSF

**Figure 1:**
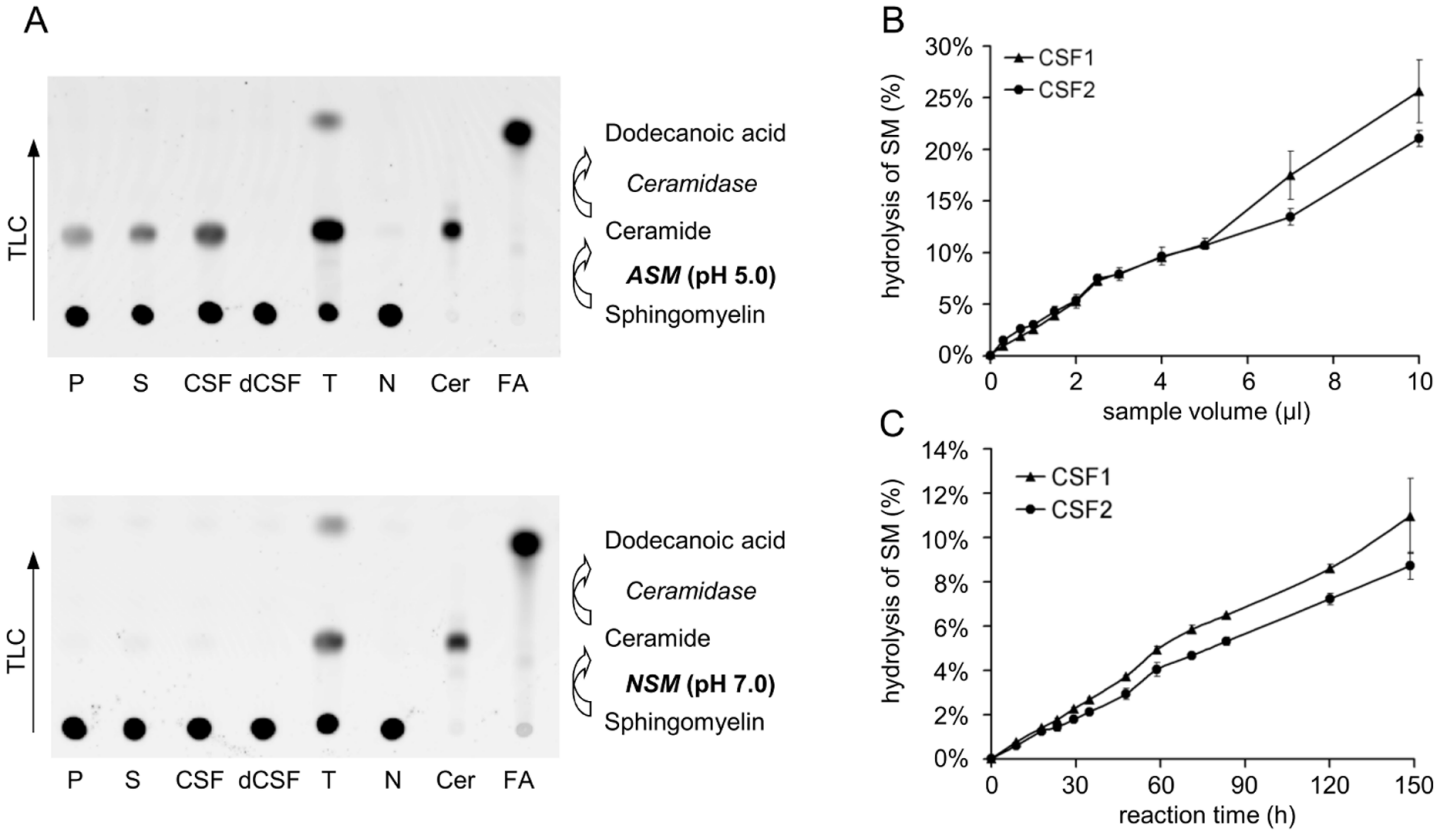
Detection of S-ASM activity in CSF and linear relationship between product, sample volume and time. A: Separation of reaction mixtures by thin layer chromatography with the fluorescent substrate BODIPY-C12-SM at the application site and the migrated fluorescent product, BODIPY-C12-ceramide, for samples of plasma (P), serum (S), tissue lysate (T), CSF in addition to heat-denatured CSF (dCSF) and water (N) as the negative control as well as ceramide (Cer) and dodecanoic acid (FA) as reference. B: Linear correlation between the amount of generated product and the applied volume. C: Linear correlation between the amount of generated product and the reaction time.

A clear signal for the hydrolysis of the fluorescent substrate BODIPY-C12-SM to BODIPY-C12-ceramide was detectable within several days of incubation of CSF samples under reaction conditions utilized for plasmatic S-ASM (Fig. 1A). The retardation factor of the resulting product, which was separated by thin layer chromatography, was identical to the value obtained by incubation of plasma, serum or tissue lysate and to that of fluorescently labeled ceramide (Fig. 1A). Thus, the hydrolysis of SM to ceramide indicates the presence of ASM in CSF. No further degradation of the BODIPY-C12-ceramide product to BODIPY-dodecanoic acid was observed following separation of the reaction mixture in a suitable solvent, suggesting the absence of acid ceramidase in CSF (Fig. 1A).

Increasing the sample volume from 0.2 to 10 µl per 100 µl of total reaction volume led to a linear increase in the amount of ceramide produced (Fig. 1B); thus, there was no inhibitory effect of volumes >3–4 µl as observed for plasmatic S-ASM (C. Mühle, unpublished), no substrate limitation and no product inhibition up to 30% conversion of SM to ceramide. The linear increase in SM hydrolysis with incubation time (up to 150 hours) indicates that the enzyme is stable at the given reaction conditions and at 37°C (Fig. 1C). Based on these data, we chose a sample volume of 1–2 µl with an incubation time of 24 hours for further experiments to efficiently utilize the precious sample material.

### Optimization of Reaction Conditions

**Figure 2:**
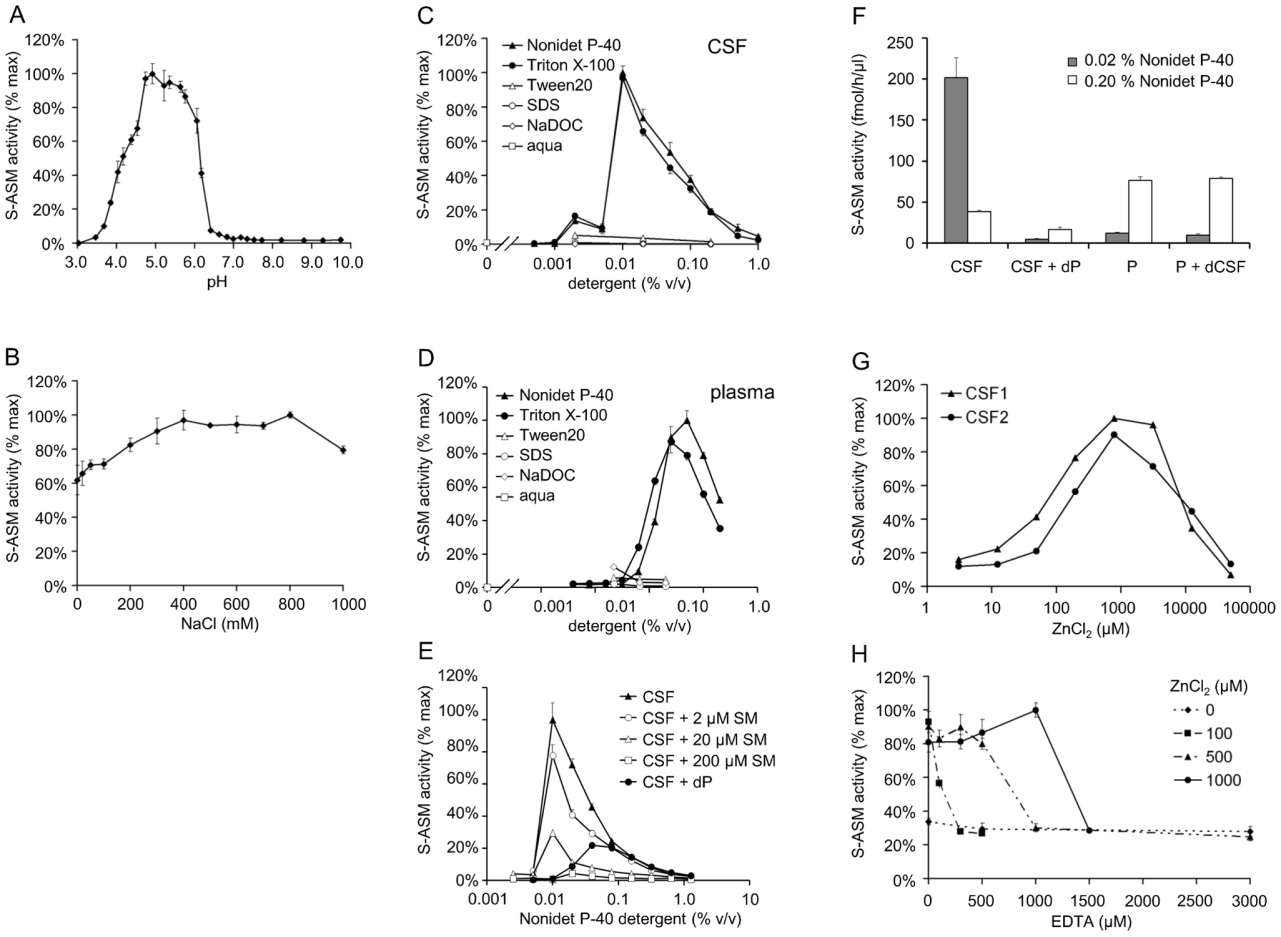
Optimization of reaction conditions for S-ASM activity in CSF. A: Activity in a series of Carmody buffers of varying pH. B: Broad optimal range for NaCl concentrations. C: Strong influence of the type and concentration of detergent (SDS, sodium dodecyl sulfate; NaDOC, sodium deoxycholate). D: Strong influence of the type and concentration of detergent on S-ASM originating from plasma for comparison. E: Dependence of S-ASM activity in CSF on the concentration of Nonidet P-40 detergent in the presence of additional bovine SM or denatured plasma (dP). F: Comparison of the effect of the detergent concentration on S-ASM from CSF and plasma in the presence of denatured plasma (dP) or CSF (dCSF), respectively. G: S-ASM dependence on addition of zinc ions. H: Very low residual S-ASM activity following addition of various amounts of EDTA.

To increase assay sensitivity, reaction conditions were optimized with respect to a number of parameters. Within the range of pH 3–10, the highest S-ASM activities were measured between 5.0–5.6. The activity decreased to less than 10% of the maximum value at pH <4 and >6.4 (Fig. 2A). These results obtained using Carmody buffers were confirmed with a set of buffers containing sodium acetate, phosphate, Tris/HCl or glycine/NaOH, respectively. No sphingolytic activity was detected even after prolonged incubation with supplemented Mg^2+^ at neutral pH, indicating the absence of the neutral sphingomyelinase [21] in CSF (Fig. 1A).

A broad optimal range of 400–800 mM was observed for the NaCl concentration (Fig. 2B). In contrast, the enzymatic activity was substantially influenced by the type and concentration of detergent (Fig. 2C). Only Nonidet P-40 and Triton X-100 increased the reaction rate whereas hydrolysis was negligible in the presence of Tween®20, sodium dodecyl sulfate or sodium deoxycholate. There was a strong inverse correlation between the enzymatic activity and the concentration of Nonidet P-40 or Triton X-100 (0.01%–1%). Nonidet P-40 at a concentration of 0.02% was chosen as the detergent for future analyses which differs from considerably higher optimal concentrations of Nonidet P-40 or Triton X-100 for S-ASM originating from plasma (Fig. 2D). Supplementation of the reaction with increasing concentrations of SM from bovine brain containing primarily stearic and nervonic acids up to 200 µM did not alter the detergent profile but resulted in a reduced hydrolytic activity towards the labeled SM substrate (Fig. 2E). Interestingly, complementation with heat-denatured plasma (1.5 µl per 100 µl reaction corresponding to approximately 6 µM SM [22]) shifted the optimum towards higher detergent concentrations (Fig. 2E). Conversely, addition of heat-denatured CSF did not exert any effect on the activity of plasmatic S-ASM (Fig. 2F). Treatment of the substrate or the final reaction with ultrasound did not influence the reaction rate.

The requirement of exogenously added Zn^2+^ for detection of ASM activity revealed that this activity originates from the secreted form rather than from released lysosomal enzyme. The optimal Zn^2+^ concentration for S-ASM activity in CSF was 0.5–1.0 mM ZnCl_2_; concentrations >10 mM had an inhibitory effect on ASM activity (Fig. 2G). In contrast to the lysosomal form that is able to retain its activity in the presence of mild zinc chelators, lack of Zn^2+^ or the addition of EDTA yielded only low residual S-ASM activities in CSF (Fig. 2H).

Using optimized assay conditions for CSF samples, we achieved a median intra-assay coefficient of variation of 8% and an inter-assay coefficient of variation of 9% (n = 5). The limit of detection (3 blank standard deviations above blank values) was 1.5 fmol/h/µl (sample volume of 1.5 µl). The lowest activity that can be reproducibly quantified in CSF with a coefficient of variation <20% was 5 fmol/h/µl, which corresponded to <2% of the normal level in control CSF samples.

### Kinetics of S-ASM in CSF

**Figure 3:**
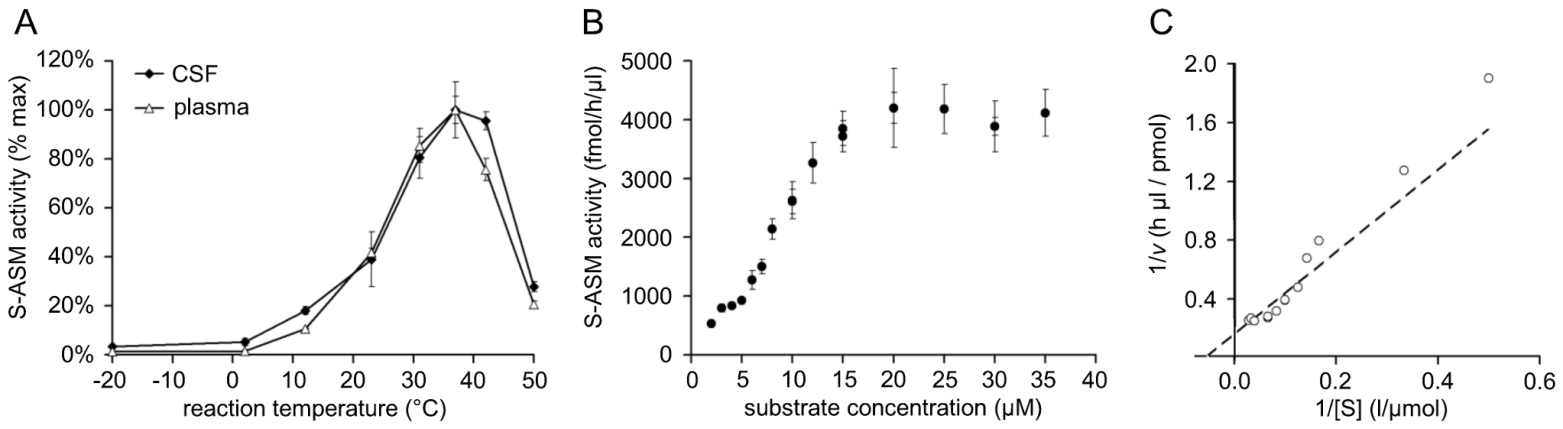
Michaelis-Menten kinetics for the reaction of S-ASM in CSF with an optimum at 37°C. A: Influence of incubation temperature on S-ASM activity in CSF. B: Dependence of S-ASM activity on the concentration of the substrate BODIPY-C12-SM under constant concentrations of DMSO (introduced by the substrate stock). C: Lineweaver-Burk plot with the line graph calculated by nonlinear regression.

The temperature profile of S-ASM from CSF resembled that of ASM originating in the plasma. As expected, both enzymes were most active at 37°C with residual activities below 30% at temperatures above 50°C or below 12°C (Fig. 3A).

A constant percent conversion rate of SM to ceramide for substrate concentrations of 0.1 µM to 10 µM suggested that the standard assay concentration of 0.6 µM SM is far below the enzyme’s saturation limit (Fig. 3B). Non-linear regression analysis using the classical model of Michaelis and Menten resulted in an apparent Km value of 20 µM. Thus, use of the ideal substrate concentration (at least 10 times the Km value for a saturated assay) is not realistic for economical and practical reasons. Supplementation of the reaction with unlabeled bovine SM decreased rather than increased the conversion of the fluorescent SM to ceramide (Fig. 2E). The reaction of S-ASM from CSF followed Michaelis-Menten kinetics with maximum activity (Vmax) at 6 pmol/h/µl (Fig. 3C). There was no evidence of cooperativity based on the shape of the graph.

### CSF Storage Conditions

**Figure 4:**
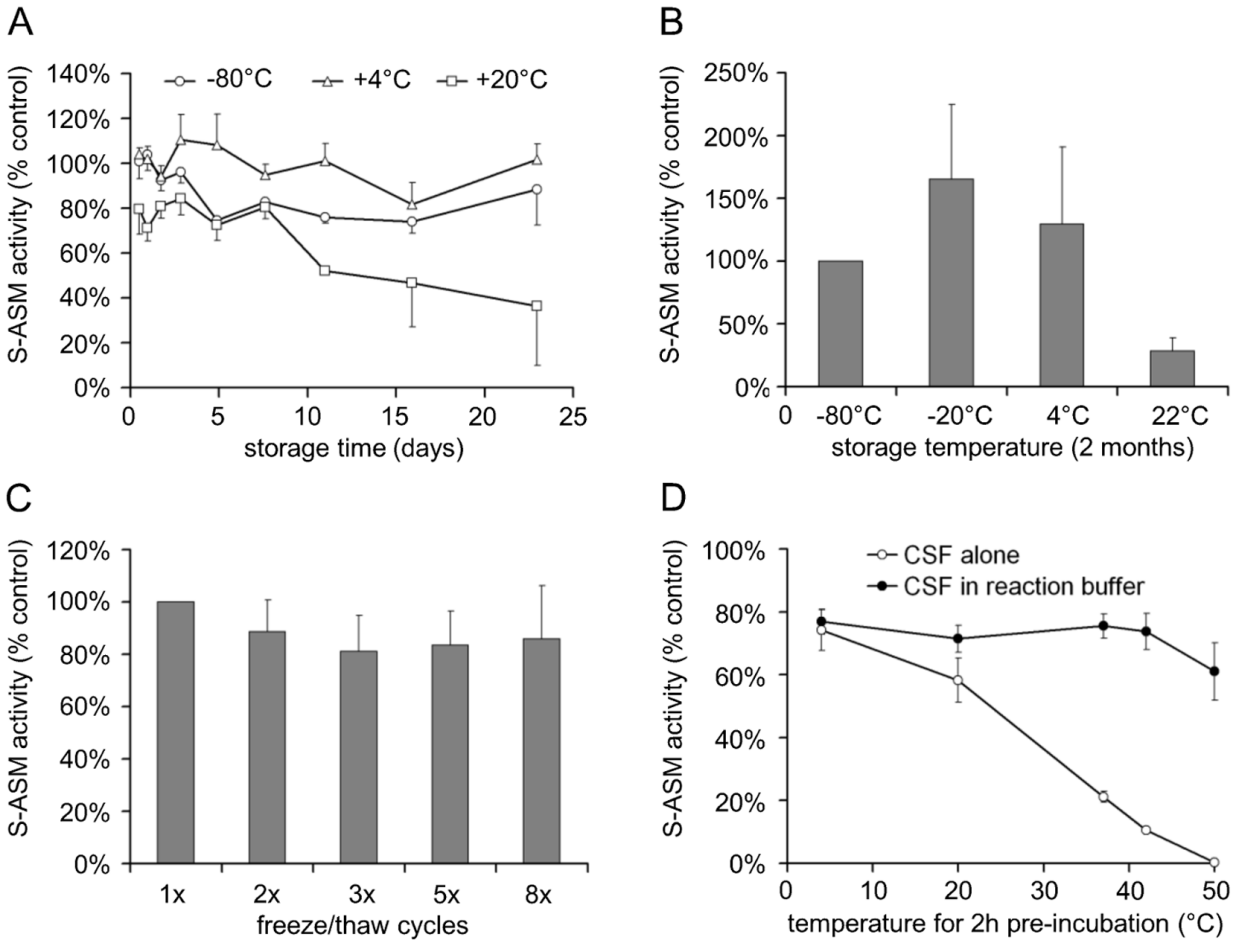
Influence of temperature and freeze/thaw cycles on S-ASM activity in CSF. A: S-ASM activity of CSF samples stored for up to three weeks at different temperatures (average of three samples). B: Increased S-ASM activity following prolonged storage of CSF samples at −20°C for two months, less change in S-ASM activity following storage at 4°C and decreased S-ASM activity after room temperature storage (average of 12 samples). C: Reduced S-ASM activity by up to 20% following three or more cycles of freezing at −80°C and short thawing at room temperature (average values of six samples). D: Heat-sensitivity of S-ASM in CSF stored at various temperatures as aliquot alone vs. preserved activity in acidic reaction buffer prior to the addition of substrate.

Sample stability is an important aspect of CSF processing and archiving. During short-term storage (up to three weeks), S-ASM activity in CSF stored at −80°C or 4°C remained nearly constant (Fig. 4A). Even at room temperature, only approximately 3% of activity was lost per day. Storage or shipment of CSF as dried spots on filter paper, however, appears to be impossible since no activity was recovered (data not shown).

Interestingly, we have found an increase in ASM activity from CSF stored at −20°C, which is similar to observations of plasma and cell lysates. During long-term storage (several months), the S-ASM activity increased considerably and variably at −20°C compared to aliquots stored at −80°C (Fig. 4B). While storage at −80°C is important, samples can be re-frozen as there is only a minor decrease in activity following several freeze-thaw cycles (Fig. 4C).

Storage of very small volume CSF aliquots (<20 µl) at room temperature or above should be avoided due to the rapid loss of S-ASM activity. In contrast, enzymatic activity was largely preserved at temperatures up to 40°C for at least 2 hours under acidic reaction conditions (Fig. 4D). This stabilizing effect is in agreement with the linear increase of the hydrolysis product over more than 5 days (Fig. 1C).

### Altered ASM Activity Levels in CSF of ASM Knock-out and ASM Transgenic Mice

**Figure 5:**
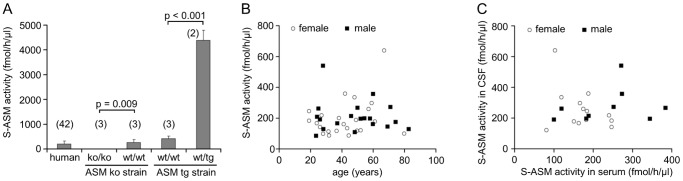
Variation of S-ASM activity in CSF from mouse models and control patients. A: Comparable activity between human and wildtype (wt/wt) mouse CSF samples, undetectable levels in ASM knock-out mice (ko/ko) and 10-fold higher activity in transgenic mice (wt/tg; number of individuals in brackets). B: Independence of S-ASM activity from the patient’s sex and age. C: No correlation between S-ASM activities in serum and CSF.

To validate the origin of the detected S-ASM activity, CSF was collected from ASM knock-out mice (defective for the expression of the ASM encoding gene *Smpd1*) and from transgenic mice (overexpressing *Smpd1*). The S-ASM activity in the CSF of wildtype sibling mice was comparable to the activity observed in humans; however, the −/− *Smpd1* knock-out mice lacked any detectable enzymatic activity. Conversely, the S-ASM activity in the CSF of transgenic mice was increased more than 10-fold compared to their wildtype siblings (Fig. 5A).

### Variation of S-ASM Activity in CSF of Patients – No Correlation with Serum S-ASM

In a pilot study, we measured the activity of S-ASM in CSF from two groups of patients classified as controls based on routine plasma and CSF parameters within the normal range (Table 1). Both groups were similar with respect to age, sex distribution, available plasma and liquor parameters except for lactate (p = 0.004, t-test). Activities of S-ASM in CSF differed to some extent between the groups but both exhibited a high variance (29% and 47%, respectively). There was no influence of age, and no difference between males and females was detected (group A: 150±39 vs. 122±36; group B: 277±112 vs. 257±136, Fig. 5B).

In general, CSF localized ASM activities were comparable to serum ASM activities at their respective optimal reaction conditions with identical substrate concentrations (58 pmol, Table 1). Importantly, no correlation between CSF and corresponding serum activities of S-ASM was observed (Pearson: r = −0.014, p = 0.952, Fig. 5C), indicating the potential utility of CSF-ASM activity as an independent biomarker.

A general linear model was used to assess the impact of all measured plasma and CSF parameters in addition to demographic data on the activity of S-ASM in CSF. None of the F values for CSF cell count, protein, albumin, lactate or plasma C reactive protein (CRP) were statistically significant to indicate an association of the covariate with the S-ASM activity in CSF (Table 1).

## Discussion

Assays for lysosomal ASM activity in cell or tissue lysates are well-established using radioactive ^14^C or fluorescent substrates with reaction times of minutes to hours. In a recent report on S-ASM in plasma [13], incubation with BODIPY-C12-SM for 24 h resulted in strong signals. Although Takahashi *et al.* determined significant enzymatic activities of ASM in various human extracellular body fluids including tear, salivary, synovial fluid and urine, they were unable to observe any ASM activity in CSF [15]. To the best of our knowledge, there have been no reports regarding CSF localized ASM activity. We were interested in determining whether prolonged hydrolysis time and optimized reaction conditions would allow the detection of sphingolytic activity in CSF.

For the first time, we report the presence of S-ASM activity in human and mouse CSF. We have established a sensitive assay using fluorescently labeled substrate to reliably detect this activity in samples as small as 0.5–2 µl of CSF. For rapid and routine ASM assays, the long incubation time (24 hours) could be reduced by increasing the substrate concentration and enhancing signal detection. We expect that separation of the ceramide product from uncleaved SM by high-performance liquid chromatography would yield comparable results. The quantification of the S-ASM activity in CSF should be equally possible by other methods such as radioactive or alternative fluorescent substrates or colorimetric assays if reaction conditions are analogously adapted for this enzyme.

Upon optimization, we have concluded that the inhibitory effect of high detergent concentrations used by Takahashi *et al*. (1% Triton X-100, and additionally 0.05% taurodeoxycholic acid from the substrate solution) in combination with a shorter incubation time could explain their failure to detect ASM in CSF despite the use of 100 µl CSF in a reaction volume of 200 µl [15]. Interestingly, the optimal detergent concentration of only about 0.01% Nonidet P-40 for the detection of S-ASM in CSF represents the most prominent difference to the reaction conditions used to assess plasmatic S-ASM (0.2% Nonidet P-40). Analysis of the enzyme’s specificity for different SM species and optimal conditions for the reverse reaction could reveal further differences between S-ASM in plasma/serum and in CSF.

Although based on an artificial fluorescently labeled substrate, the reaction followed Michaelis-Menten kinetics with an apparent Km value of 20 µM which is in agreement with published values for similar ASM species: 77 µM for soluble ASM in human epidermis [23], 25 µM for purified placental ASM [24] and 65 µM for ASM from *Bacillus cereus* [25]. The substrate concentration (0.6 µM) used in our assay was far below the saturation level; this requires an exact substrate dilution, parallel reference samples or the preparation of large reaction mixtures that proved to be stable at 4°C for several months.

Several characteristics including the optimal pH and Zn^2+^ dependence for the measured sphingolytic activity in human CSF support the notion, that the observed activity is the result of S-ASM encoded by *SMPD1*. The most convincing evidence is provided by the significantly increased activity in CSF of transgenic ASM mice carrying an additional *Smpd1* copy and by the lack of activity in CSF of ASM knock-out mice. Similarly, Niemann-Pick disease patients are expected to show reduced ASM activity in CSF. While human vascular endothelial cells are known to secrete high amounts of ASM in cell culture studies [26], the ability of ependymal cells, which are responsible for most of the daily CSF production, to secrete the ASM enzyme is not yet known. Although neutral sphingomyelinase activities in rat brain, liver and kidney tissues are approximately 20-fold higher compared to ASM activities [27], no secretory neutral sphingomyelinase was detectable in our CSF samples using BODIPY-C12-SM.

The remarkable stability of ASM activity in CSF stored at 4°C or room temperature for several days allows intermediate storage and simple shipping of study samples if the expected deviations from normal values were relatively large. Long-term storage, however, requires a temperature of −80°C because storage at −20°C leads to an activation of ASM in CSF. This effect has been also observed for plasmatic S-ASM (C. Mühle, unpublished). Moreover, activities of human recombinant ASM in harvested cell media following storage at −20°C for several weeks prior to the purification of the enzyme were substantially increased. This activating effect was attributed to the loss of a single free cysteine residue (C629) by chemical modification during the freezing process [28]. While a series of parameters including storage temperature and repeated freeze/thaw cycles have already been evaluated, further criteria analogous to those of the Alzheimer’s Biomarkers Standardization Initiative for pre-analytical aspects of CSF biomarker testing [29] should be analyzed, such as the effects of fasting, intraday variance or type of collection tubes.

In our pilot study, we did not observed any influence of sex or age on ASM activity in CSF. In contrast, a significant increase in sphingolipid metabolic enzyme activities in kidney, liver and brain has been reported during the development and aging of rats [27]. Compared to postnatal day 1, the ASM activity in the brain had increased 2.5-fold by day 180 and reached 5-fold levels by day 720. Additional studies describe an accumulation of ceramide and sphingosine in the liver and brain with age, supporting increased sphingomyelinase and ceramidase activities [30]. Further experiments are required to determine whether the age-dependent change of enzymatic ASM activity is restricted to brain tissue or to lysosomal ASM or whether an effect of age on ASM activity in CSF would be evident in a larger study.

Increased ASM activity has been associated with the stress response, inflammation and systemic immune challenges and is thought to be an early event in the development of Alzheimer’s disease (AD) with inflammation-induced deposits of amyloid precursor protein [31]. Although no correlation between CSF localized ASM and plasmatic CRP or any of the other CSF parameters analyzed was evident in our small control group, we would expect elevated S-ASM levels in the CSF of patients with AD or inflammatory disorders of the brain where CSF samples are routinely evaluated for diagnosis. This assumption is supported by the observation of an association of higher baseline ratios of plasma SM to ceramide – a possible indication of lower ASM activity - with less cognitive progression in AD [32]. Although elevated levels of ceramide in the CSF of patients with Alzheimer’s disease [33] could also be attributed to an inhibition of ceramide-metabolizing enzymes, significantly increased levels of total ceramide and of individual ceramide species in brains of patients with AD corresponded to an upregulated transcription of ASM [34]. Moreover, an activation of membrane-associated ASM was detected in AD brains with significant SM reductions and ceramide elevations and was found to highly correlate with the levels of Abeta and phosphorylated tau protein [11]. If similar alterations were observed for S-ASM activity in accessible CSF as seen in the brain of patients with AD, this parameter could be integrated into our interpretation algorithm [35], [36] and supplement established CSF biomarkers to improve sensitivity, specificity and diagnostic accuracy of the algorithm.

Therapeutic interventions to reduce increased ASM levels could include the administration of direct ASM inhibitors [37] or functional inhibitors of ASM [38]. Many antidepressant and antipsychotic drugs functionally inhibit lysosomal ASM in cell culture models [39]. It is unknown whether these drugs also directly affect the S-ASM levels, particularly the enzyme found in CSF, or whether they indirectly influence its levels via shunting of the common precursor protein [26], for example, or via alternative splice variants [40]. If so, CSF samples from patients treated with drugs that act as functional inhibitors of ASM would be expected to have altered S-ASM activities in CSF compared to unmedicated patients.

Decreased activities of S-ASM are less likely to cause pathological conditions in the central nervous system. Patients with the less severe Niemann-Pick disease type B, who retain more than 10% residual ASM activity, suffer from accumulation of SM in viscera but are spared from neurological symptoms [8]. Moreover, in a combined knock-out/transgenic mouse model, stable expression of low levels of lysosomal ASM was sufficient to preserve the function of the central nervous system despite the absence of secretory enzyme [41].

In summary, we have established a sensitive enzyme assay and we provide evidence for the presence of S-ASM activity in human and mouse CSF. Our extensive biochemical characterization and quantification in human control CSF samples establish the basis for further studies to identify disease conditions with altered S-ASM activity in CSF and to assess its value as a biomarker for disease type, severity, progress or therapeutic success, which would be independent of serum or plasma S-ASM activity levels.
